# High-Stability Bi-Layer Films Incorporated with Liposomes @Anthocyanin/Carrageenan/Agar for Shrimp Freshness Monitoring

**DOI:** 10.3390/foods12040732

**Published:** 2023-02-08

**Authors:** Junjun Zhang, Yan Yang, Jianing Zhang, Jiyong Shi, Li Liu, Xiaowei Huang, Wenjun Song, Zhihua Li, Xiaobo Zou, Megan Povey

**Affiliations:** 1 Agricultural Product Processing and Storage Laboratory, School of Food and Biological Engineering, Jiangsu University, Zhenjiang 212013, China; 2 China Light Industry Key Laboratory of Food Intelligent Detection & Processing, School of Food and Biological Engineering, Jiangsu University, Zhenjiang 212013, China; 3 China Light Industry Engineering Technology Research Center of Central Kitchen Intelligent Equipment, School of Food and Biological Engineering, Jiangsu University, Zhenjiang 212013, China; 4 International Joint Research Laboratory of Intelligent Agriculture and Agri-Products Processing, Jiangsu Education Department, Jiangsu University, Zhenjiang 212013, China; 5 Collaborative Innovation Center for Modern Grain Circulation and Safety, College of Food Science and Engineering, Nanjing University of Finance and Economics, Nanjing 210023, China; 6 School of Food Science and Nutrition, University of Leeds, Leeds LS2 9JT, UK

**Keywords:** liposomes, high stability, freshness, bi-layer indicator

## Abstract

High-stability bi-layer films were prepared by incorporating anthocyanin-loaded liposomes into carrageenan and agar (A-CBAL) for non-destructive shrimp freshness monitoring. The encapsulation efficiency of the anthocyanin-loaded liposomes increased from 36.06% to 46.99% with an increasing ratio of lecithin. The water vapor transmission (WVP) of the A-CBAL films, with a value of 2.32 × 10^−7^ g · m^−1^ · h^−1^ · pa^−1^, was lower than that of the film with free anthocyanins (A-CBA). The exudation rate of the A-CBA film reached 100% at pH 7 and pH 9 after 50 min, while the A-CBAL films slowed down to a value lower than 45%. The encapsulation of anthocyanins slightly decreased the ammonia sensitivity. Finally, the bi-layer films with liposomes successfully monitored shrimp freshness with visible color changes to the naked eye. These results indicated that films with anthocyanin-loaded liposomes have potential applications in high-humidity environments.

## 1. Introduction

The spoilage of meat products, which is extremely harmful and destructive, significantly increases the risk to human health [1]. Therefore, it is necessary to detect meat freshness. In the past, total volatile basic nitrogen (TVB-N) was widely regarded as a useful method for meat freshness monitoring using the Kjeldahl method [2]. However, it is destructive to samples and time-consuming. In recent years, more studies have been interested in intelligent packaging systems for ‘’on-package” tracing in real-time. Intelligent food packaging is an effective tool for monitoring food conditions for consumers through intuitive changes. Meat corruption produces volatile amines, which results in an alkaline packaging environment. Therefore, pH indicator films, as a kind of intelligent sensor, have garnered wide attention because they can reflect freshness information through visual color changes. As a natural extract, anthocyanin presents visible color changes at different pH values and has been used in intelligent pH indicator films in recent years [3]. For instance, Zhang et al. successfully developed a novel film based on a mulberry anthocyanin extract for fish freshness monitoring [4]. However, most reported indicator films are based only on individual anthocyanins in the film-forming matrices, which makes them easily degraded in harsh environments (such as light and temperature). In addition, water-soluble free anthocyanins easily leak out from the film matrix in a high-humidity environment, thereby affecting the indicator stability. This instability creates a barrier that limits the use of intelligent film in practical food packaging. Therefore, it is essential to use an effective method to improve the stability of anthocyanin indicators.

Nano/microencapsulation technology in particular has been verified as a useful method to enhance the stability of anthocyanins. In terms of encapsulation technology, liposomes are popularly prepared with phospholipids, oils, and different solvents [5]. The central aqueous cavity of liposomes can be used to improve the stability of hydrophilic active ingredients and increase bioavailability [6]. Liposomes are attractive because they can encapsulate anthocyanins without changing their structure. The prepared anthocyanin elderberry extract-loaded liposome has the highest encapsulation efficiency of 69% and storage stability [7]. The retention rate of anthocyanins in milk was effectively improved with liposomes prepared by Chi et al. [8]. Up to now, few researchers have reported the use of liposomes to encapsulate free anthocyanins in intelligent packaging films. 

Compared with single-layer biopolymer films, bi-layer films have shown excellent mechanical properties and stability [9,10]. One layer serves as an indicator layer containing anthocyanins and the other as a protective layer. Agar, one of the most widely promising agents, is applied in food packaging films due to its good gelling properties and excellent film-forming materials [11]. Carrageenan, a natural biopolymer, is widely studied as a packaging film matrix due to its high gelling capacity [12]. Because of the stronger hydrogen bond interactions between their highly polar hydroxyl groups, agar and carrageenan are used together to improve their mechanical properties. 

Therefore, in this study, a liposome was formed by encapsulating a butterfly bean flower anthocyanin extract (BA) in soybean lecithin, which was then added to carrageenan to develop as an indicator layer. In addition, agar was used as the protective layer of a bi-layer indicator to monitor shrimp freshness. The particle size, zeta potential, morphology, and encapsulation efficiency of the liposome were initially analyzed. The bi-layer indicator film was determined with Fourier transform infrared spectroscopy (FTIR), scanning electron microscopy (SEM), and its mechanical properties. Moreover, the temperature stability and the response sensitivity to pH solutions and ammonia of the bi-layer films were investigated before evaluating their application for shrimp freshness monitoring.

## 2. Materials and Methods

### 2.1. Materials

The dried butterfly bean flower calyxes and fresh shrimp were obtained from the Zhenjiang Darunfa supermarket. Agar, ammonia, ethanol, potassium chloride, and hydrochloric acid were purchased from Sinopharm Chemical Reagent Co., Ltd. Citric acid, sodium acetate, and sodium dihydrogen citrate were bought from Jiangsu Thorpe Group Co., Ltd. (Zhenjiang, China) Carrageenan, glycerol, and disodium hydrogen phosphate were obtained from Jiangsu Chentong Chemical Co., Ltd. (Zhenjiang, China). Soybean lecithin, cholesterol, TritonX-100, and Tween80 were acquired from Zhenjiang Huadong Chemical Glass Co., Ltd. (Zhenjiang, China).

### 2.2. Preparation and Characterization of Anthocyanin-Loaded Liposomes

#### 2.2.1. Extraction of Butterfly Bean Flower Anthocyanin

The butterfly bean flower anthocyanin (BA) was obtained according to a previous study [13]. The dried butterfly bean flower calyxes were crushed into a powder. Then, approximately 100 g of the powder was macerated with 1 L of 75% ethanol for 3 h at 60 °C. The solvent extraction solution was obtained using a centrifuge at 3000 r/min for 6 min. After that, the anthocyanin concentrated solution was obtained to remove the ethanol solvent using a rotary evaporator (RE-200A, SHANGHAI YARONG biochemistry instrument factory, China) at 50 °C for 2 h. Finally, the concentrated solution was dried in a vacuum freeze-dryer to obtain a BA powder.

#### 2.2.2. Preparation of BA-Loaded Liposomes

The BA-loaded liposomes (BALs) were prepared using the ethanol injection high-pressure homogenization method with some modifications [14]. Lecithin (0.2%, 0.5%, and 1%), cholesterol (0.2%), and Tween 80 (0.24%) were dissolved in an ethanol solution. The BA solution (0.2%) was prepared in an acetate buffer solution (pH 3.5, 0.05 mol/L). To obtain crude milky liposomes, the BA solution was quickly injected into lecithin mixtures of different concentrations and stirred vigorously for 30 min. Next, the crude liposomes were homogenized using a high-pressure homogenizer (AH-BASIC, Antos Nano Technology Co., Ltd., Suzhou, China) at 20,000 psi for 5 cycles. Then, the cooling solution was passed through a 0.25 μm extruder, and the solvent was removed via rotary evaporation (SY-4000, Shanghai Yarong Co., Ltd., Shanghai, China) to obtain concentrated BALs with different concentrations of lecithin at 0.2%, 0.5%, and 1% (BAL1, BAL2, and BAL3).

#### 2.2.3. Characterization of the Liposomes

The average particle size, zeta potential, and polydispersity index (PDI) of the liposomes were evaluated with the dynamic light scattering technique using a Zeta-sizer Nano ZS (Malvern, Worcestershire, UK). The microstructure of the liposomes was observed using an optical microscope (4XC-W, Jinanchenda, Jinan, China).

The encapsulation efficiency (EE) was measured according to the literature with some modifications [15]. Solutions of various concentrations (0.5, 1.0, 1., 2.0, and 2.5 μL/mL) of the anthocyanin were dissolved in a buffer solution (pH 6.86), and the absorbance was measured at 620 nm. The standard curve of the anthocyanin was analyzed as the equation Y = 0.221x + 0.010 (R^2^ = 0.9981). Then, a certain amount of BALs was immersed in a buffer solution and centrifuged at 8000 rpm for 20 min. Free anthocyanins were isolated from the supernatant, and their absorbance was measured at 620 nm. Then, the concentration of free anthocyanins was calculated with the standard curve. Finally, the EE of the anthocyanin was obtained as follows:

(1) $$\mathrm{EE}(\%)=\frac{\mathrm{Total}\;\mathrm{anthocyananins}-\mathrm{Free}\;\mathrm{anthocyanins}}{\mathrm{Total}\;\mathrm{anthocyanins}}$$

#### 2.2.4. The Color of BAL in Different pH Solutions

The absorbance of the BA and BAL solutions at different pH values was measured in the range of 450 nm to 700 nm using a UV-visible spectrophotometer (TU10CS, Beijing General Analytical Instrument, Beijing, China).

### 2.3. Preparation of the Bi-Layer Indicator Films

The bi-layer films were prepared with two individual solvent casting methods. Firstly, 2 g of agar was stirred in 100 mL of distilled water for 2 h at 100 °C. Then, an agar hydrogel was formed as the outer layer by cooling the plastic Petri dish at room temperature. Secondly, 2 g of carrageenan was stirred in 100 mL of water with 2% glycerin for 1 h at 85 °C. After cooling at 65 °C, free anthocyanins and different groups of BALs (BAL1, BAL2, and BAL3, each containing 20 mg of the anthocyanin) were added to the above carrageenan solution. The solutions were thoroughly stirred at 65 °C for 30 min. Finally, the carrageenan solutions containing free anthocyanins and liposomes were dispersed onto the agar protective layer and dried in an oven for 24 h at 35 °C, and the bi-layer films were obtained and termed as the A-CBA, A-CBAL1, A-CBAL2, and A-CBAL3 films, respectively.

### 2.4. Structural Properties of the Bi-Layer Films

#### 2.4.1. Microstructure

The cross-sections of the bi-layer films were performed with a JSM-3400 (JEOL Ltd., Tokyo, Japan) at an accelerating voltage of 10 keV. Prior to observation, the samples were divided into small pieces and vertically adhered to an aluminum stub with a thin layer of gold.

#### 2.4.2. FTIR Analysis

The FTIR spectra of the films and film-forming materials were measured using a Nicolet 50 spectrometer in the attenuated total reflection mode at 4000–525 cm^−1^ with a resolution of 4 cm^−1^ (Thermo Scientific, Waltham, MA, USA).

### 2.5. Determination of Physical Properties of Bi-Layer Films

#### 2.5.1. Mechanical Properties

The thickness of the films was determined using a Mitutoyo digital micrometer (Tester Sangyo Co., Ltd., Saitama, Japan). The mechanical properties were defined using a TA-XT Plus texture analyzer (Stable Micro Systems, Godalming, UK). The films were cut into 20 × 60 mm pieces with an initial distance of 40 mm and a proper tensile speed of 0.6 mm/s [16].

#### 2.5.2. Water Vapor Transmission (WVP) Results

*WVP* was determined using the standard gravimetric method of ASTM E96-05. The films were covered on top of a 50 mL centrifuge tube with 20 mL of water and stored in a desiccator. *WVP* was analyzed according to the centrifuge tube weight every 12 h for 5 days and calculated with the following formula:

(2) $$WVP=\frac{\Delta m\times d}{S\times \Delta P\times t}$$

where *d* is the average thickness (mm); *S* is the effective permeation area of the film (m^2^); ∆*m* is the mass of water permeation (g); *t* is the interval time(s); and ∆*P* is the pressure difference between the 2 sides of the film (3179 Pa).

#### 2.5.3. Color Appearance and Opacity

The colors of the bi-layer films were measured using a portable scanner (G4050, HP, USA) and then expressed as L*, a*, and b* values. The opacity of the films was recorded with a UV-vis spectrophotometer at 200 to 800 nm. The opacity formula was as follows [11]:

(3) $$Opacity=\frac{{Abs}_{600}}{d}$$

where *Abs*_600_ is the absorbance at 600 nm, and *d* is the average thickness (mm).

### 2.6. Color Stability of Bi-Layer Films

In order to measure the stability, the films were kept at 4 °C or 25 °C at 2-day intervals within 14 days using a portable scanner. The calculation of color changes (Δ*E*) was as follows:

(4) $$\Delta E=\sqrt{{\left(L-L_{0}\right)}^{2}+{\left(a-a_{0}\right)}^{2}+{\left(b-b_{0}\right)}^{2}}$$

where *L*, *a*, and *b* are the color values of the films at storage time; *L*_0_, *a*_0_, and *b*_0_ are the initial color values.

### 2.7. Color Response and the Leaching Rate under Different pH Buffers of Films

The film samples were immersed in plastic Petri dishes containing 15 mL of buffer solutions (2–10). During the different time intervals, the exudation rate was determined by calculating the concentration of the anthocyanin leaching solution, and the color response was captured using a camera at the beginning time.

### 2.8. Color Response to Ammonia of Bi-Layer Films

Each of the bi-layer films was placed into the middle-upper layer of a sealed, homemade acrylic box (500 mL). An aqueous ammonia solution was injected into the bottom of each box with 0.1 mL of different concentrations at 0–200 μM, and the color changes were determined using a CM2300 spectrophotometer [17]. The digital values were also expressed as color changes (Δ*E*).

### 2.9. Application in Shrimp Freshness Detection of Bi-Layer Films

According to the determinations of the film results, the A-CBA and A-CBAL2 films were used as shrimp freshness indicators. An amount of 50 g of fresh shrimp was placed inside a sealed packing box (700 mL), whose inner surface was attached to a film at 4 °C for 96 h. The color of each film was obtained using a CM2300 spectrophotometer every 12 h. The total volatile basic nitrogen (TVB-N) value was determined according to the method of Zhang et al. [17].

### 2.10. Data Analysis

All tests were repeated three times with mean ± standard deviation results. Duncan’s test was used to analyze the data in SPSS software (Version 21, IBM SPSS Inc, New York, NY, USA), and the differences were considered significant if *p* < 0.05.

## 3. Results and Discussion

### 3.1. Characterization of the BAL Liposomes

The results of different liposomes with average particle sizes, Zeta potentials, PDI values, microstructures, and EE values are shown in Table 1. With the addition of lecithin, the average particle sizes of the liposomes obviously increased from 131.39 nm to 311.42 nm, which was attributed to the amount of hydrogen and van der Waals force between the anthocyanins and lecithin [18]. Zeta potential is an important parameter to characterize the stability of liposomes. The higher value of Zeta potential, the greater repulsion strength required to settle and coagulate liposomes [19]. The Zeta potentials of BAL1 and BAL2 were −48.23 mV and −40.16 mV, respectively, indicating the stable dispersion of liposome particles in the solution. PDI is an index that reflects the particle size distribution [20]. The smaller the PDI, the better the regularity of dispersion of the particles. A PDI < 0.4 indicates a homogenous particle size distribution in the system [21]. With the addition of lecithin, the PDI increased from 23.96% to 29.51%, indicating the heterogeneous size distribution. This was consistent with the Zeta potential results. These structure formations can also be observed in the microstructures of the multi-compartmental but obvious core–shell structures. Thus, the EE increased from 36.06% to 46.99% with the increasing ratio of lecithin. The above results indicated that the ratio of lecithin was one of the key factors in the characterization of anthocyanin-loaded liposomes.

### 3.2. The pH Response of Anthocyanin-Loaded Liposomes

**Figure 1:**
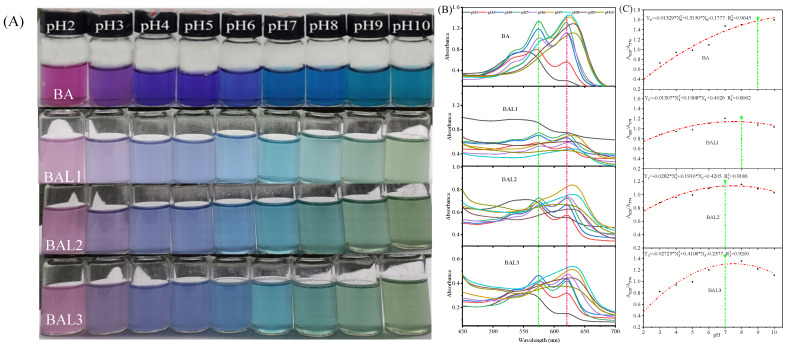
Color (**A**) and ultraviolet-visible spectra (**B**), and the ratio of A_620_ and A_574_ (**C**) of BA and BAL at different pH values.

As shown in Figure 1A, both anthocyanins and liposomes showed obvious color changes in different pH values. The color of BA changed from pink to purple, then blue, and finally blue-green. The color of BAL1 changed from pink to purple-green, then cyan, and finally green. The different color changes of anthocyanin were caused by structural transformations, which were found in a previous study [13]. In fact, the different color changes between BA and BAL were attributed to the cavity structure of liposomes, which decreases the structural transformation rate in anthocyanins [22]. As shown in Figure 1B, 2 characteristic absorption peaks can be observed around 574 nm and 620 nm for BA and BAL. At pH 2, the absorption peak of BA was at 552 nm and gradually red-shifted to 574 nm at pH 3–8. With the pH increasing to 9–10, the absorption peak disappeared due to the destroyed structure of the anthocyanin molecular center ring under strong alkaline conditions [23]. The response mechanism of BAL to pH was consistent with that of the anthocyanin solution. However, the peak at 574 nm disappeared at pH 8 for the BAL1 spectrum while occurring at pH 7 for the BAL2 and BAL3 spectrums, respectively. This is mainly because of the encapsulation difference. The ratio of A_620_ to A_574_ reflected the shift changes of the absorption peaks in Figure 1C. This was clearly observed in the variation of the maximum values of the BA and BAL spectra. The above results showed that the coloration degree of the solution obviously decreased after being encapsulated by liposomes, while the color sensor function of the anthocyanins was not hindered.

### 3.3. The Structural Analysis of the Bi-Layer Films

#### 3.3.1. SEM Analysis of Indicator Films

**Figure 2:**
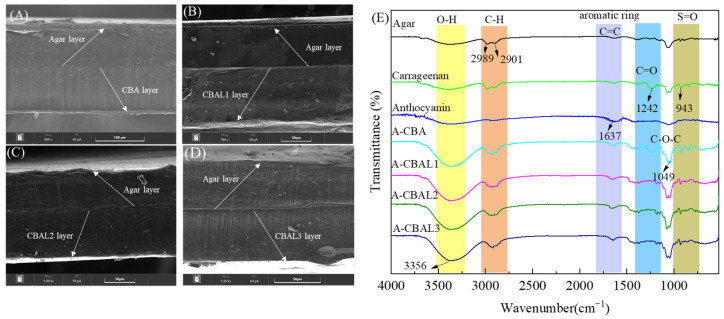
Cross-section SEM images of A-CBA (**A**), A-CBAL1 (**B**), A-CBAL2 (**C**), and A-CBAL3 films (**D**), and FTIR spectra (**E**) of colorimetric films.

The film compatibility can be observed in the cross-section of a bi-layer film. As can be seen in Figure 2, all the films presented an obvious two-layer structure, which was attributed to the hydrogel thermal irreversibility processes between agar and carrageenan. Meanwhile, hydrogen bonding, cross-linked agar, and carrageenan prevented the bi-layer films from separating. The agar outer layers appeared relatively uniform except for the parts that were contaminated by the inner anthocyanin layers. In Figure 2A, the A-CBA film with free anthocyanins displays a homogeneous and compact structure. Compared with the free anthocyanins, the liposomes with hydrophobic structures of the A-CBAL film caused a reduction in the cross-linking between the film-forming solution and water molecules. Therefore, the liposomes in the film-forming matrix presented a lower homogeneous dispersion. However, there were no obvious differences between the A-CBAL films, indicating that the anthocyanin encapsulation of liposomes hardly presented a negative effect on the film morphologies. Importantly, the above results indicated that bi-layer films were satisfactorily prepared.

#### 3.3.2. FTIR Analysis of Indicator Films

The absorption peaks of the FIIR spectra of the film-forming materials and the bi-layer films are shown in Figure 2E. The band at 3356 cm^−1^ corresponds to the OH stretching vibration of the hydroxyl structure. It occurred in all the spectra but with lower peak intensity changes [24]. The peaks at 2289 and 2901 cm^−1^ were due to the C-H and -CH_2_ stretching vibrations of alkane groups [25]. The absorption band that appears at 1637 cm^−1^ of the anthocyanins was ascribed to the C=C stretching from the aromatic ring frame of the butterfly bean flower anthocyanin, which is related to the flavonoid fingerprint spectra and was also found in all the bi-layer films [26]. The absorption peaks of carrageenan at 1242 and 943 cm^−1^ were associated with the C=O of the glycoside bond and the S=O of the sulfate ester group, respectively [27]. The other major band at 1049 cm^−1^ was attributed to the C-O-C stretching vibration of the conjugated carbonyl group [28]. In the case of the A-CBAL films, all the peaks presented similar positions with minor intensity changes to the control film (A-CBA). The results indicated that there was no chemical interaction between the liposomes and anthocyanins.

### 3.4. Physical Performance Analysis

#### 3.4.1. Appearance and Opacity Analysis Results

The intuitive packaging color appearance can easily affect the application efficacy of an indicator film. As presented in Table 2, there were no obvious differences in the L* values of A-CBAL films with different lecithin ratios but they exhibited slightly higher values than the A-CBA film, indicating that liposome films have higher brightness. The decreased negative a* and b* values reflect the lower greenness and blueness strengths of the films with liposomes, which could be attributed to the yellowish color of the liposomes. Therefore, the blue of anthocyanin was covered after being encapsulated by liposomes, increasing the opacity.

#### 3.4.2. Thickness and WVP Analysis Results

**Figure 3:**
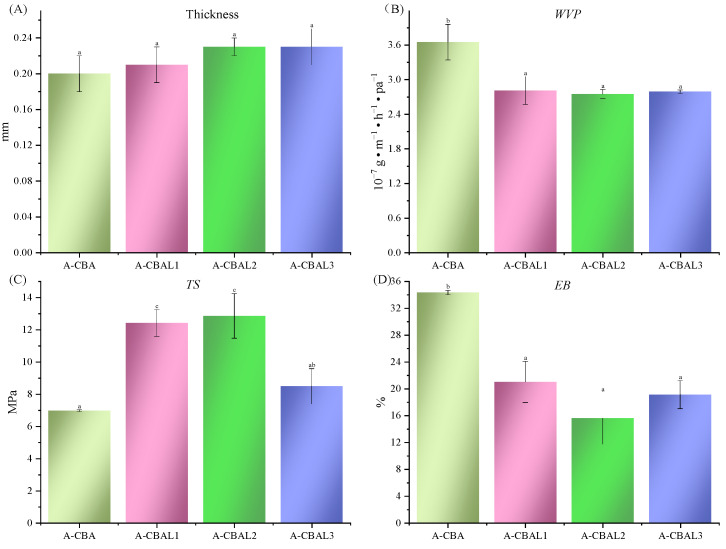
Thicknesses (**A**), *WVP* values (**B**), and mechanical properties with *TS* (**C**) and *EB* values (**D**) of the bi-layer films. Characters represent a significant difference (*p* < 0.05).

As illustrated in Figure 3A, the thicknesses of all the indicator films were not significantly different. The considerable index evaluates whether the packaging quality is WVP, which can represent the ability to block external water vapor of a film. As summarized in Figure 3B, the WVP values of all the A-CBAL films were significantly lower than that of the A-CBA film. It may be that lecithin had hydrophobic tails, which reduced the hydrophilicity of the indicator film. Therefore, the liposome films reflected higher water vapor resistance. However, the films with different lecithin ratios had little difference between them, and the maximum values did not exceed 2.32 × 10^−7^ g · m^−1^ · h^−1^ · pa^−1^.

#### 3.4.3. Mechanical Properties

Excellent TS and EB values can improve the protection performance of food packaging materials during food transportation periods. Each of the A-CBAL films had a significantly higher TS value and a lower EB value than the A-CBA film. This was probably attributed to the stronger intramolecular chemical bonding force and intermolecular force (van der Waals force and hydrogen bond) between liposomes and the film-forming materials than free anthocyanins [29]. The A-CBAL2 film simulated the maximum TS value with a value of 12.42 MPa, and that of the A-CBAL3 film gradually decreased to 5.81 MPa due to liposome instability. However, there were no significant differences in the EB values of the three A-CBAL films, that is, the change in the lecithin ratio did not destroy the crystal structure of the film-forming matrix.

### 3.5. Exudation Rate and Color Response of Indicator Films to pH Solution

**Figure 4:**
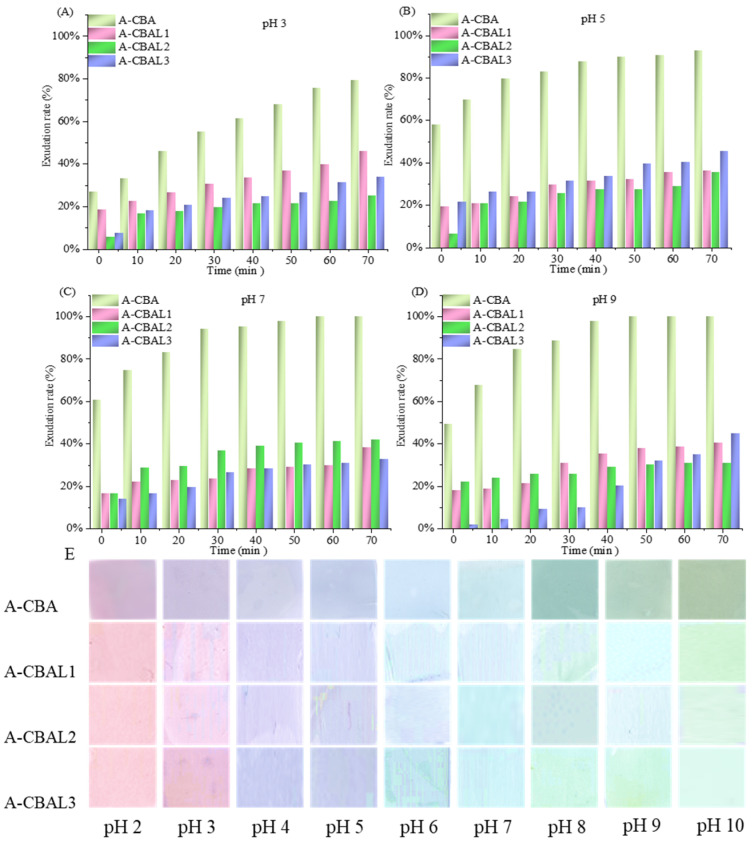
The exudation rates of bi-layer films at pH 3 (**A**), pH 5 (**B**), pH 7 (**C**), and pH 9 (**D**), and the color of the films at pH 2-10 (**E**).

The issue of anthocyanin leaking out from films causes the failure of the indicator function. The pH behavior of the films differed noticeably, as seen in Figure 4. The exudation rates of bi-layer film with free anthocyanins (A-CBA) exuded rapidly, reaching 80% at pH 2 after 70 min. In addition, the exudation rate of the A-CBA film reached 100% at pH 7 and pH 9 after 50 min, due to the higher degradation of anthocyanins under the alkaline environment [30]. Thus, the films with anthocyanin-loaded liposomes (A-CBAL) slowed down the exudation rate by no more than 45%. However, there was no correlation between the anthocyanin exudation rate and the ratio of lecithin in liposomes. In conclusion, the liposomes enhanced anthocyanin encapsulation, which can improve the stability of an indicator film in a high-humidity environment.

Figure 4E shows the color responses of the indicator films at different pH values. With pH increases, the color of the A-CBAL films changed from pink to purple and then gradually tended toward yellowish green. It can be verified that the color changes of the indicator films were consistent with anthocyanin-loaded liposome solutions, but with different degrees of coloration. However, compared with the A-CBA film, the response chrominance of the A-CBAL films decreased, which corresponded to the color appearance results in the encapsulation of liposomes. The encapsulation hindered the coloration of the butterfly bean flower anthocyanin. With the addition of lecithin, the coloration of the indicator films decreased, but they still presented visible color changes. As a result, in high-humidity food packaging, our bi-layer film with anthocyanin-load liposomes can be used as a pH indicator.

### 3.6. Color Stability of the Bi-Layer Films

**Figure 5:**
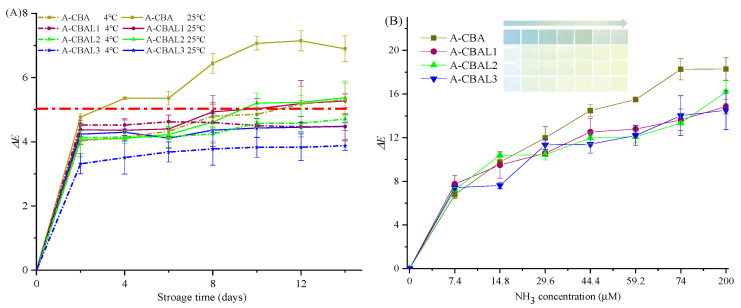
Color changes of the films stored at 4 °C (**A**) and 20 °C (**B**) for 20 d.

The storage stability of the indicator films was determined under 4 °C and 25 °C, respectively. Generally, when the Δ*E* value of an indicator is no more than five, it will be difficult to notice with the naked eye [31]. As can be seen from Figure 5A, each of the bi-layer films presented higher stability with a lower Δ*E* value at 4 °C within 14 days. Thus, the film with free anthocyanins was not stable on the 4th day at 25 °C with an Δ*E* value of 5.35. The values of the A-CBAL1 and A-CBAL2 films were greater than 5 on the 10th day. At 25 °C, the films were more easily able to form a ring-opened chalcone structure with color changes [32]. The Δ*E* value of the A-CBAL3 film was 4.48 on the 14th day. This was because more radio lecithin with high encapsulation could protect free anthocyanins from external intrusion.

### 3.7. Response Analysis of Indicator Films to Ammonia

The indicator films exposed to ammonia with different Δ*E* values can be seen in Figure 5B. The colors changed from baby blue to light green and then to yellow green with the increase in ammonia concentration. Moreover, the Δ*E* values were consistent with the visible colors of the films. All of the indicator films had the same variation trend but with some differences. Compared with A-CBAL films, the film with free anthocyanins presented the highest color changes, with an Δ*E* value of 18.29. The encapsulation of anthocyanins decreased their ammonia reactive ability. Even so, the A-CBAL2 film also presented visible color changes with Δ*E* values above 14.28. However, there were slight differences between the films with different liposomes. As a result, a film containing anthocyanin-loaded liposomes also has the potential to serve as a food freshness indicator.

The *Lab* values (A–E), *b* values (F), Δ*E* (G), and color changes of the films (H) with the concentration of ammonia.

### 3.8. Application on Monitoring Shrimp Freshness of Bi-Layer Indicator Film

**Figure 6:**
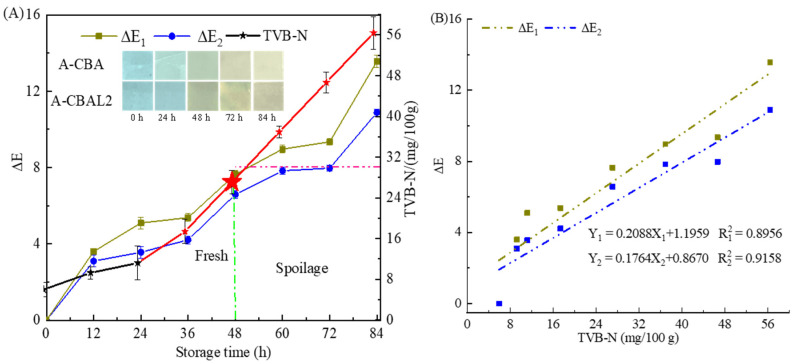
The TVB-N of shrimp and Δ*E* of the films (**A**). Δ*E*_1_ is A-CBA and Δ*E*_2_ is A-CBAL2 at the storage time. Correlation relation between TVB-N and Δ*E* (**B**). The illustration is the color change of films during storage.

In this study, the A-CBA and A-CBAL2 films were used to monitor shrimp freshness at 4 °C. As shown in Figure 6, the Δ*E* of the indicator films and the TVB-N of the shrimp exhibited a similar increasing trend during the storage time. The TVB-N increased to 11.20 mg/100 g and the corresponding Δ*E*_2_ was 3.56 for the A-CBAL2 film, with little color change in the first 24 h. Then, the films changed from blue to dark green-yellow with an Δ*E*_2_ value of 6.58, and the Δ*E*_1_ value of the A-CBA film was 7.63 after 48 h. The *TVB-N* was 27.08 mg/100 g at 48 h. The freshness of shrimp was still approved because the legislation limiting level of *TVB-N* is 30 mg/100 g in Seawater shrimp (GB2733-2015). After 60 h, the *TVB-N* increased to 36.93 mg/100 g, which was spoiled, and the Δ*E* increased dramatically due to the shrimp’s deeper putrefaction. The Δ*E*_1_ value was 8.96 for the A-CBA film with a deepened yellow color, and the Δ*E*_2_ value was 7.83 with a light yellowish color. The color of the film with liposomes was lower than that of the film with free anthocyanin, which may be attributed to the encapsulation of the anthocyanin by liposomes, which reduced the color response sensitivity of the anthocyanin. Meanwhile, the correlation analysis between the Δ*E* of the bi-layer film and the *TVB-N* followed a linear model. For the A-CBA film, the coefficient was 0.8956, and for the A-CBAL2 film, it was 0.9158 (Figure 6B). Therefore, the film with anthocyanin-loaded liposomes can also be used as a good indicator for the detection of the putrefaction period of shrimp.

## 4. Conclusions

In this study, free anthocyanins and anthocyanin-loaded liposomes were added to carrageenan as the sensor layer of the bi-layer films, respectively, and agar was the outer protective layer. Different ratios of lecithin were used to design the butterfly bean flower anthocyanin extraction into liposomes, and their characterization was investigated. Then the structure, mechanical physical properties (such as TS, EB, and WVP), stability, pH, and ammonia sensitivity of the bi-layer films were individually analyzed with different ratios of lecithin in liposomes. The SEM and FT-IR results indicated that the bi-layer films were satisfactorily prepared via hydrogen bonding interactions. The films with anthocyanin-loaded liposomes had significantly higher TS values and lower EB values than that with free anthocyanins. Importantly, the films with liposomes had a positive effect on the stability of the indicator films in high-humidity environments but slightly decreased the pH and ammonia sensitivity. Finally, the application on the shrimp verified that the bi-layer film can be used as an indicator of meat freshness. However, the encapsulation of anthocyanins by liposomes delayed the sensitivity of the film. Therefore, future exploration could focus on a higher sensitivity method based on liposomes.

## Figures and Tables

**Table 1:** The sizes, Zeta potentials, PDI values, EE values, and microscope pictures of the liposomes.

	Average Particle Size/nm	Zeta Potential/mV	PDI/%	Microscope	EE/%
BAL1	131.39 ± 5.12 ^a^	−49.01 ± 1.52 ^c^	23.96 ± 2.03 ^a^	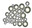	36.06 ± 1.87 ^a^
BAL2	153.49 ± 10.53 ^b^	−43.16 ± 0.88 ^b^	27.57 ± 3.53 ^bc^	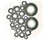	44.28 ± 4.25 ^b^
BAL3	311.42 ± 15.68 ^c^	−19.96 ± 2.31 ^a^	29.51 ± 4.25 ^c^	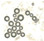	46.99 ± 6.17 ^bc^

Note: the superscripted characters a, b, c represent significant differences (*p* < 0.05).

**Table 2:** The colors and opacity values of the films.

Film	L*	a*	b*	Opacity	Appearance
A-CBA	84.82 ± 0.42 ^a^	−10.52 ± 1.89 ^c^	−7.37 ± 1.01 ^d^	15.08 ± 0.86 ^a^	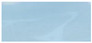
A-CBAL1	92.65 ± 1.31 ^b^	−1.54 ± 0.35 ^a^	−1.08 ± 0.11 ^a^	32.39 ± 1.18 ^c^	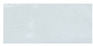
A-CBAL2	89.61 ± 1.05 ^b^	−2.45 ± 0.56 ^b^	−1.59 ± 0.40 ^ab^	27.72 ± 2.45 ^b^	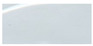
A-CBAL3	90.17 ± 0.56 ^b^	−2.25 ± 0.31 ^b^	−3.11 ± 0.79 ^c^	26.02 ± 1.82 ^b^	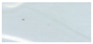

Note: the superscripted characters of a, b, c, d represent significant differences (*p* < 0.05).

## Data Availability

The original contributions presented in the study are included in the article; further inquiries can be directed to the corresponding author.
